# Editorial: Endotyping and phenotyping prematurity and its complications

**DOI:** 10.3389/fped.2023.1217530

**Published:** 2023-06-06

**Authors:** Maria Pierro, Roy Philip, Laurent Renesme, Eduardo Villamor

**Affiliations:** ^1^ Neonatal Intensive Care Unit, Bufalini Hospital, Cesena, AUSL Romagna, Italy; ^2^ Neonatal Intensive Care Unit, University Maternity Hospital Limerick and University of Limerick School of Medicine, Limerick, Ireland; ^3^ Neonatal Intensive Care Unit, Children’s Hospital of Eastern Ontario, Ottawa, ON, Canada; ^4^ MosaKids Children’s Hospital, Maastricht University Medical Center (MUMC+), School for Oncology and Reproduction (GROW), Maastricht University, Maastricht, Netherlands

**Keywords:** prematurity, endotype, phenotype, targeted therapy, precision medicine

**Editorial on the Research Topic** Endotyping and phenotyping prematurity and its complications

The mechanisms triggering preterm birth are multifold and largely unexplored. However, evidence is increasingly supporting the hypothesis that the pathophysiological conditions eliciting preterm birth play a major role in the clinical picture and the development of complications of prematurity (1). Most of the complications of prematurity involve different forms of organ injury, expressing themselves as different clinical phenotypes. These phenotypes are highly influenced by the pathological intrauterine environment that manifests themselves as endotypes of prematurity (1). The term endotype refers to “a subtype of a condition, which is defined by a distinct functional or pathophysiological mechanism” (2). A consensus on the different endotypes of prematurity has yet to be reached. Two major pathways leading to very preterm birth have been identified so far: the infection/inflammation endotype and the dysfunctional placentation endotype (3). The infection/inflammation endotype includes chorioamnionitis, preterm labor and rupture of membranes and cervical insufficiency. The dysfunctional placentation endotype includes placental malperfusion, hypertensive disorders of pregnancy (HDP) and/or intrauterine growth restriction. A better understanding of the role of the prematurity endotypes may facilitate the design of more targeted therapeutic and prognostic approaches to the preterm population.

The objective of this research topic was to deepen the knowledge about the association between endotypes of prematurity and phenotypes of complications of prematurity. This topic covered different neonatal organs and systems as they can all be altered by the endotypes of prematurity.

Prenatal environment may affect brain maturation and the process leading to brain injury in preterm infants. The periventricular white matter damage, defined as periventricular leukomalacia (PVL), is the most common form of non-hemorrhagic brain injury in preterm infants (4). PVL is a result of various antenatal, intrapartum, and postnatal insults to the developing brain and represents the most common cause of preterm birth-related cerebral palsy in childhood (4). A better mechanistic understanding of the origins of PVL may help develop preventive and therapeutic strategies. Su et al. used a propensity score matching analysis to evaluate the effect of HDP on clinical outcomes of extremely preterm or extremely low birth weight (EP/ELBW) infants. In this case-control study the incidence of PVL in the offspring of mothers suffering from HDP was significantly increased. The authors demonstrated that, after matching the six major covariates including sex, gestational age, birth weight, antenatal steroids administration and methods of conception, HDP increased the risk of PVL in EP/ELBW infants however had no significant effect on the survival rate at discharge, or the incidence of other complications. The possible explanation for this result may be related to the hypoxic intrauterine environment typical of HDP. Hypoxia is one the major upstream mechanisms of PVL (4). In the case of maternal HDP, the fetus is exposed to chronic hypoxia in the intrauterine environment (5). Placental hypoxia leads to fetal oligodendrocyte dysmaturation, resulting in altered myelination, ultimately inducing PVL (6).

Similarly to brain maturation, the nature, timing, and extent of prenatal environment may alter fetal and neonatal endothelial function (7, 8). Changes in the luminal surface, oxidative stress, growth factors imbalance, and dysregulation of permeability and vascular tone are the leading causes of endothelial dysfunction in preterm infants (9). Preterm birth impairs numerous endothelial pathways with potentially severe early and late neonatal adverse outcomes. Amelio et al. reviewed the current knowledge on endothelial dysfunction in relation to the etiology of preterm birth. The type and extent of endothelial dysfunction may vary according to the intrauterine environment. The paper summarizes the available evidence on the type of endothelial damage in the infectious/inflammatory and dysfunctional placentation endotypes of prematurity, focusing on their molecular peculiar features, biomarkers, and clinical impact. In the given context, advancing endothelial characterization could be a promising way to provide patient-tailored care to the most vulnerable newborns.

Individual studies have suggested that prenatal conditions such as chorioamnionitis or HDP may also affect the therapeutic response to pharmacological agents in preterm infants (10). In particular, response to cyclooxygenase (COX) inhibitors, used to treat patent ductus arteriosus (PDA), may vary depending on fetal programming induced by the intrauterine enviroment. Who, when and how to treat PDA is a challenge for the neonatologist. Multiple factors including gestational age, birth weight, day of life at treatment may influence the effectiveness of PDA treatment. In addition, intrauterine conditions may play a role in the neonatal response to ductal therapy. The meta-analysis from Gonzalez-Luis et al. showed a significant association between exposure to HDP and increased rate of pharmacological closure of PDA. In contrast, neither chorioamnionitis nor being small for gestational age were significantly associated with the response to therapy. Subgroup analyses showed that the higher response to COX inhibitors in the HDP group was significant for indomethacin but not for ibuprofen or for the studies using both drugs. However, meta-regression showed that this difference between the drugs was not statistically significant. These data suggest that the pathologic condition that triggers prematurity may alter the response to pharmacological treatment of PDA and may help address uncertainties around PDA management.

Bronchopulmonary dysplasia (BPD), is a preterm-related chronic lung disease (11) that may involve airways, alveoli, vessels, interstitium, and lymphatic system, giving rise to different clinical phenotypes, that may be difficult to unveil in clinical practice (12, 13). Cardiopulmonary ultrasound (CPUS) may allow a better understanding of the cardiorespiratory challenges that the neonatologists face on a daily basis, tailoring the diagnostic approach and targeting treatments for BPD (14). The case report from Bruno et al. described four different cardiopulmonary ultrasound patterns of evolving and established chronic lung disease of prematurity and the consequent therapeutic choices. In the reported cases, the histological examination of the placental material and the identification of the endotypes of prematurity were consistent with the CPUS patterns. This approach, if confirmed in prospective studies, may guide the personalized management of infants suffering from evolving and established BPD, optimizing the chances of success of the therapies and at the same time reducing the risk of exposure to inadequate and potentially harmful drugs.

Recently, the public health mitigation measures during COVID-19 lockdowns around the world offered an unprecedented and unique “nature's experiment” influencing a multitude of host and environmental factors on the feto-maternal unit, and multiple studies suggested changes to preterm birth rates of EP/ELBW population (15). Calvert et al. analysed 52 million births from 26 countries to evaluate changes in preterm birth during the lockdowns (iPOP study) and concluded that large number preterm births were averted globally, warranting further research into causal pathways (16).

A better insight of the relationship between endotypes and phenotypes of prematurity may help target treatments in the view of personalized medicine and in the context of developments in artificial intelligence, potentially improving the outcome of the fragile preterm population.

## References

[1] Mir IN Leon R Chalak LF. Placental origins of neonatal diseases: toward a precision medicine approach. Pediatr Res. (2021) 89(2):377–83. 10.1038/s41390-020-01293-6 33288874

[2] Lötvall J Akdis CA Bacharier LB Bjermer L Casale TB Custovic A Asthma endotypes: a new approach to classification of disease entities within the asthma syndrome. J Allergy Clin Immunol. (2011) 127(2):355–60. 10.1016/j.jaci.2010.11.037 21281866

[3] McElrath TF Hecht JL Dammann O Boggess K Onderdonk A G Markenson Pregnancy disorders that lead to delivery before the 28th week of gestation: an epidemiologic approach to classification. Am J Epidemiol. (2008) 168(9):980–9. 10.1093/aje/kwn202 18756014 PMC2720771

[4] Schneider J Miller SP. Preterm brain injury: white matter injury. Handb Clin Neurol. (2019) 162:155–72. 10.1016/B978-0-444-64029-1.00007-2 31324309

[5] Mol BWJ Roberts CT Thangaratinam S Magee LA de Groot CJM Hofmeyr GJ. Pre-eclampsia. Lancet. (2016) 387(10022):999–1011. 10.1016/S0140-6736(15)00070-7 26342729

[6] Gumusoglu SB Chilukuri ASS Santillan DA Santillan MK Stevens HE. Neurodevelopmental outcomes of prenatal preeclampsia exposure. Trends Neurosci. (2020) 43(4):253–68. 10.1016/j.tins.2020.02.003 32209456 PMC7170230

[7] D'Alquen D Kramer BW Seidenspinner S Marx A Berg D Groneck P Activation of umbilical cord endothelial cells and fetal inflammatory response in preterm infants with chorioamnionitis and funisitis. Pediatr Res. (2005) 57(2):263–9. 10.1203/01.PDR.0000148713.48218.86 15611353

[8] Yu GZ Reilly S Lewandowski AJ Aye CYL Simpson LJ Newton L Neonatal micro-RNA profile determines endothelial function in offspring of hypertensive pregnancies. Hypertension. (2018) 72(4):937–45. 10.1161/HYPERTENSIONAHA.118.11343 30287978 PMC6166786

[9] Mezu-Ndubuisi OJ Maheshwari A. Role of the endothelium in neonatal diseases. Newborn. (2022) 1(1):44–57. 10.5005/jp-journals-11002-0025 35754998 PMC9217741

[10] Kim ES Kim EK Won Choi C Kim HS Kim B Choi JH Intrauterine inflammation as a risk factor for persistent ductus arteriosus patency after cyclooxygenase inhibition in extremely low birth weight infants. J Pediatr. (2010) 157(5):745–50.e1. 10.1016/j.jpeds.2010.05.020 20598319

[11] Pierro M Van Mechelen K van Westering-Kroon E Villamor-Martínez E Villamor E. Endotypes of prematurity and phenotypes of bronchopulmonary dysplasia: toward personalized neonatology. J Pers Med. (2022) 12(5). 10.3390/jpm12050687 35629108 PMC9143617

[12] Pierro M Villamor-Martinez E van Westering-Kroon E Alvarez-Fuente M Abman SH Villamor E. Association of the dysfunctional placentation endotype of prematurity with bronchopulmonary dysplasia: a systematic review, meta-analysis and meta-regression. Thorax. (2022) 77(3):268–75. 10.1136/thoraxjnl-2020-216485 34301740 PMC8867288

[13] Villamor-Martinez E Álvarez-Fuente M Ghazi AMT Degraeuwe P Zimmermann LJI Kramer BW Association of chorioamnionitis with bronchopulmonary dysplasia among preterm infants: a systematic review, meta-analysis, and metaregression. JAMA Netw Open. (2019) 2(11):e1914611. 10.1001/jamanetworkopen.2019.14611 31693123 PMC6865274

[14] Ciarmoli E Storti E Cangemi J Leone A Pierro M. Use of cardio-pulmonary ultrasound in the neonatal intensive care unit. Children. (2023) 10(3):462. 10.3390/children10030462 36980020 PMC10047372

[15] Philip RK Purtill H Reidy E Daly M Imcha M McGrath D Unprecedented reduction in births of very low birth weight (VLBW) and extremely low birth weight (ELBW) infants during the COVID-19 lockdown in Ireland: a ‘natural experiment’ allowing analysis of data from the prior two decades. BMJ Global Health. (2020) 5(9):e003075. 10.1136/bmjgh-2020-003075 PMC7528371 32999054

[16] Calvert C Brockway M(M) Zoega H Miller JE Been JV Kofi A Changes in preterm birth and stillbirth during COVID-19 lockdowns in 26 countries. Nat Hum Behav. (2023) 7:529–44. 10.1038/s41562-023-01522-y 36849590 PMC10129868

